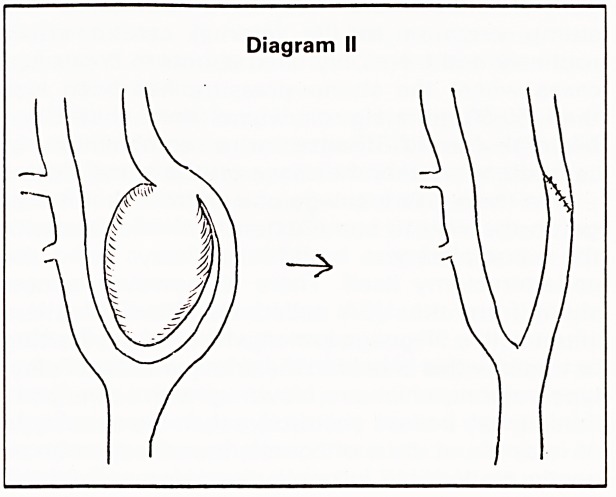# Carotid Surgery in a District General Hospital

**Published:** 1981

**Authors:** John Fairgrieve

**Affiliations:** Consultant Surgeon, Cheltenham General Hospital Paper read to the S.W. Surgical Club, Cheltenham

## Abstract

The carotid surgical experience of Cheltenham General Hospital over a 13 year period (1968-81) is presented. This includes 42 operations for stenosis, and 12 further operations for carotid body tumour, carotid aneurysm, subclavian steal syndrome and trauma to the internal carotid artery. The operative techniques and complications are briefly discussed and reasons advanced for a more agressive approach to the problems of extra-cerebral carotid disease in this country.


### Carotid Surgery in a District General Hospital

**John Fairgrieve**
Consultant Surgeon, Cheltenham General Hospital
Paper read to the S.W. Surgical Club, Cheltenham

**SUMMARY**

The carotid surgical experience of Cheltenham General Hospital over a 13 year period (1968-81) is presented. This includes 42 operations for stenosis, and 12 further operations for carotid body tumour, carotid aneurysm, subclavian steal syndrome and trauma to the internal carotid artery. The operative techniques and complications are briefly discussed and reasons advanced for a more agressive approach to the problems of extra-cerebral carotid disease in this country.

**DISCUSSION**

It has been estimated that there are 500,000 strokes annually in the USA resulting in some 200,000 deaths. 60% of these strokes are due to atheromatous brain infarction and in addition to the mortality, strokes also carry risks of severe disability, loss of independence and chronic hospitalisation.[1]

If the population of the USA is reckoned as some 200 million, i.e. approximately 4 times that of the UK, we, in Britain, can expect to have some 125,000 strokes annually with perhaps 50,000 deaths due to this cause. If we accept that perhaps half of these strokes are due to extracerebral carotid disease, and it may be more, it follows that there are some 25,000 patients with carotid bifurcation disease at risk in this country in any one year. Many of these patients would be suitable for carotid endarterectomy if their symptomatology was recognised by their GPs and if they were subsequently referred to Vascular Units for investigation and treatment. Sadly, this is not yet the case, and only a tiny fraction of this large group of patients is, in fact, referred partly because of ignorance and partly, I suspect, because our physician colleagues have yet to be convinced that the operation in the right hands is a safe one, and they accordingly rely on Aspirin therapy.

The mortality of carotid endarterectomy should, according to Jessie Thomson[2]in the USA be no more than 2-4% if the results are to be acceptable. His own results in a series of 1300 patients have been 4.9% for patients who have had a partial stroke previously, 1.2% for patients with TIA's and 0% for patients with asymptomatic bruits and his more recent results are even better than this. Less experienced surgeons may not be able to achieve such excellent results, but we can, I believe, carry out carotid surgery reasonably safely and with an acceptable mortality.

The most important role of carotid endarterectomy is in the prevention of strokes, as 35-40% of patients with untreated TIAs will go on to have frank strokes within 5 years.[3]

In the USA, according to Thomson, carotid endarterectomy is the most frequently performed peripheral vascular operation but this is certainly not the case in the UK. In the first ten months of this year in Cheltenham so far we have only done 10 carotid endarterectomies compared with 45 aortic grafts and 21 femoro-popliteal grafts. Just how many are done in this country? In 1979 Peter Morris[4] from Oxford circularised 152 members of the British Vascular Surgical Society and only 77 of these were performing carotid endarterectomies at all. Of these only 24 Surgeons performed more than 10 operations per year, and only 2 more than 50 per year. At least 6 Neurosurgeons also performed the operation, and 2 of these did more than 30 operations per year.

It seems, therefore, that we are lagging behind very badly compared with the USA and this should not be the case when we remember that the first recorded endarterectomy was performed in this country by Eastcott[5] in 1954 and that the operation has been a well established one for the last 20 years or more.

We, in Cheltenham, have only a limited, though gradually increasing experience of carotid endarterectomy and I think it would be invidious of me, if not impertinent, to present my own views when several of you probably have a far greater experience. Nevertheless, there are a number of District General Hospitals in the South West where

*no* carotid surgery is undertaken and I thought you might be interested to hear briefly of our carotid experience over the last 10 years or so.

**Table I**

**Cheltenham Carotid Experience (1969-81)**

1. Carotid endarterectomy:
   42 operations – 39 unilateral
   3 bilateral
2. Carotid body tumours:
   4 patients
3. Carotid aneurysms:
   2 patients – 1 true
   1 false
4. Subclavian Steal Syndrome: 5 cases
   Carotid – subclavian bypass – 3 patients
   Innominate endarterectomy – 2 patients
5. Trauma to internal carotid artery in petrous temporal bone:
   1 patient

### CAROTID STENOSIS

As regards the operation of carotid endarterectomy, we have used the more cosmetic skin crease incision rather than the orthodox incision along the anterior border of sternomastoid and I have not found this disadvantageous as regards access.

About half the cases, i.e. the early ones, had the operation done under local anaesthetic using cervical plexus block and phenoperidine, but in the last 5 years or so we have done them under general anaesthesia. We have monitored the stump pressure in the internal carotid artery routinely and have only used shunts in 6 selected cases where the stump pressure has been less than 50-60 mm Hg or where there has been bilateral carotid disease or a combination of carotid and vertebral disease on the same side.

The main disadvantage of a shunt is that it may get in the way to some extent and interfere with the completeness and accuracy of the endarterectomy itself. There is, however, a new shunt from the USA called the Prewlitt-Inahara shunt with a 9F guage lumen which we are starting to try out – this is held in the artery with two Foley-type baloons which are blown up with saline and I think it may be less obstructive than the usual type of inlay shunt. It is, of course, manditory to be as gentle as possible in one's dissection around the carotid bulb and to avoid dislodging debris from the surface of the plaque and so initiating an embolic stroke. We have not used patches on the artery in any of our cases.

Our main post-operative problem has undoubtedly been the rather unpredictable swings in blood pressure that occur. We have injected 1% Lignocaine routinely subadventially at the carotid bifurcation and this quickly corrects any bradycardia or hypotension at the time, but post-operatively the effect takes some hours to wear off which may not be a good thing when the carotid sinus nerve is needed to function normally afterwards. Certainly we have had a number of cases where the post-operative blood pressures has risen to dangerous heights and required Arfonad or nitroprusside for its control.

Another danger, of course, in these cases is arteriography, with the risk of dislodgement of debris from the atheromatous plaque and we have had one case of permanent unilateral blindness due to this complication. We are very fortunate in having Roger Baird in Bristol with his interest in pulsed Doppler imaging of the carotid artery[6] which is a valuable non-invasive method of assessing carotid stenosis and I would like to thank him for his help in a number of cases. Despite these investigations, there are some patients on whom the decision to operate has to be taken on the symptomatology alone as neither arteriography nor Doppler imaging may show significant stenosis or irregularity. A minor Doppler flow disturbance may be the only indication of an ulcerating plaque causing micro-embolisation.

Progressive stroke is, of course, a contra-indication to operation and in our own fatal case, the stroke occurred on the morning of operation and was not immediately recognised. The outcome of the case might well have been the same whether the operation had been performed or not, but the increased perfusion pressure following endarterectomy is likely to have serious consequences in an area of cerebral infarction.

### CAROTID BODY TUMOURS

Of our four chemodectomas, three have been in the carotid body and one was a glomus jugulare tumour which presented partly as a cervical swelling and partly as a retropharyngeal tumour.

The carotid body tumours have all had characteristic arteriograms displaying the widened carotid bifurcation (goblet sign) and increased vascularity. At operation the carotid vessels are fused with the tumour but can be dissected off

**Diagram I**

without too much difficulty in benign cases especially is saline and adrenaline is infiltrated around the adventitia of the vessels. We have not encountered a malignant carotid body tumour but this would probably require resection and replacement with a vein graft as shown in Diagram I.[7] Histologically, the appearances of the benign and malignant tumours are almost identical, and only spread to lymph nodes in the neck or distant metastases allow the distinction to be made.

### CAROTID ANEURYSMS

Of our two carotid aneurysms, the false one followed a radical attempt to remove a recurrent and infected squamous cell carcinoma of the neck. Following a secondary haemorrhage this was successfully repaired using a vein patch, a technique that we have found useful in arterial repair under a number of different circumstances, e.g. damage to the femoral artery during a Trendelenberg operation, and for repair of a popliteal A-V fistula following menisectomy.

The true carotid aneurysm which was a saccular aneurysm of the internal carotid artery was resected without the use of a shunt, and an oblique end-to-end anastomsis using 6/0 Prolene was performed (Diagram II).

### SUBCLAVIAN STEAL SYNDROME

Of the five cases of subclavian steal syndrome we have operated on, two have been treated by innominate endarterectomy and three by a cross-neck carotid-subclavian bypass, e.g. from the left common carotid to the right subclavian artery distal to the block. I prefer the latter operation which is relatively simple and it also avoids having to split the sternum or perform a difficult endarterectomy because of limited access. We did, however, tragically lose one patient several years ago when an inlay shunt in the common carotid artery was sucked into the aorta and became impacted with one end in the innominate artery and the other end tripping the aortic valve. The shunt was successfully removed later but unfortunately the patient subsequently died of respiratory complications.

### TRAUMA TO INTERNAL CAROTID ARTERY

We have also had one unusual case of accidental injury to the internal carotid artery in the petrous temporal bone[8] which occurred in the course of radical surgery to remove an invasive squamous cell carcinoma of the ear and resulted in serious haemorrhage which could only be controlled by packing.

An orthodox repair of the artery was technically impossible, an alternative method using an on-lay vein patch, held in position by a moulded bone-

**Diagram II**

cement stent, was used instead. This successfully controlled the bleeding and the patient made an uncomplicated post-operative recovery.

Long term patency of the internal carotid artery has been confirmed in this case by pulsed Doppler angiography, and the stent was subsequently extruded two and a half years later leaving an apparently tumour-free cavity.

**Table II**

**Complications**

1. **Carotid endarterectomy:**

   2 deaths – 1 progressive stroke
   – 1 cerebral embolus – hemiplegia

   3 nerve palsies – X neuropraxia – hoarse voice
   – VII recurrent branch (submandibular)
   – post. auricular nerve – numbness behind ear

   3 post-operative TIAs – all minor and reversible

   1 haematoma – requiring evacuation

2. **Carotid-Subclavian Bypass**

   1 death – lost shunt –
   late death from respiratory failure

### CONCLUSION

At the present time relatively few operations for carotid stenosis are performed in this country, and patients at risk often remain undiagnosed until too late and are seldom referred to Vascular Units for investigation and surgery.

The place of Aspirin therapy in the prevention of atheromatous cerebral disease awaits the outcome of trials in progress at the present time, but meanwhile it is to be hoped that the advent of non-invasive methods of investigation, and safer surgical techniques and case selection will lead to an increased number of patients with carotid disease being referred for surgery.

### REFERENCES

1. TAYLOR, G. W., Postgraduate Course on Cerebrovascular Disease – Presentation. International Vascular Symposium, London 1981.
2. THOMPSON, J. E., Postgraduate Course on Cerebrovascular Disease – Results. International Vascular Symposium, London 1981.
3. THOMPSON, J. E., Postgraduate Course on Cerebrovascular Disease – Results. International Vascular Symposium, London 1981.
4. MORRIS, P. J., Summary of Carotid Endarterectomy Data for the UK for 1979. Personal communication.
5. EASTCOTT, H. H. G., PICKERING, G. W. and ROB, C., Reconstruction of Internal Carotid Artery in a Patient with intermittent attacks of Hemiplegia. *Lancet,* **2,** 994, 1954.
6. LUSBY, R. J., MACHLEDER, H. I., JEANS, W., SKIDMORE, R., WOODCOCK, J. P., CLIFFORD, P. C. and BAIRD, R. N., Vessel wall and blood flow dynamics in arterial disease. *Phil.Trans.R.Soc.,* London B 294, 231-39, 1981.
7. DENT, T. L., THOMPSON, N. W. and FRY, W. J., Carotid Body Tumours. *Surgery,* 1976, 80, 365-72.
8. FAIRGRIEVE, J., HARDINGHAM, M. and BENJAMIN, P. J., Repair of the intra-osseous portion of the internal carotid artery using bone cement. *Br.J.Surg.,* 1981, 68, 666-67.

## Figures and Tables

**Diagram I f1:**
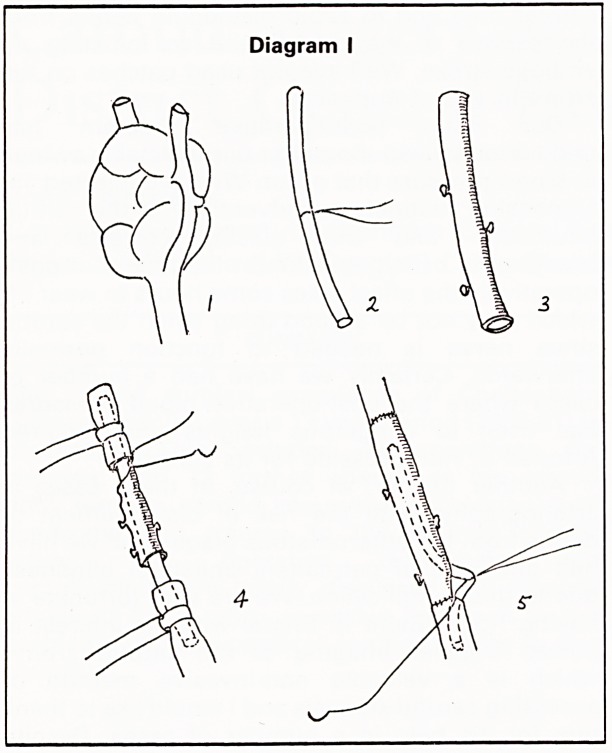


**Diagram II f2:**